# Comparative effects of *Curcuma longa *and curcumin on paraquat-induced systemic and lung oxidative stress and inflammation in rats

**DOI:** 10.22038/AJP.2022.19713

**Published:** 2022

**Authors:** Seyedeh Zahra Ghasemi, Arghavan Memarzia, Sepideh Behrouz, Zahra Gholamnezhad, Mohammad Hossein Boskabady

**Affiliations:** 1 *Applied Biomedical Research Center, Mashhad University of Medical Sciences, Mashhad, Iran*; 2 *Department of Physiology, School of Medicine, Mashhad University of Medical Sciences, Mashhad, Iran*; 3 *Student Research Committee, Mashhad University of Medical Sciences, Mashhad, Iran*

**Keywords:** Paraquat, Curcuma longa, Curcumin, Oxidative stress, Inflammation, Lung, WBC

## Abstract

**Objective:**

Comparative effect of *Curcuma longa *(*C. longa*) ethanolic extract and curcumin on paraquat (PQ)-induced systemic and lung oxidative stress and inflammation were evaluated in the present study.

**Materials and Methods:**

Control animals were exposed to normal saline and PQ group to 54 mg/m^3^ PQ aerosols (8 times, each time for 30 min). Treatment groups were exposed to PQ and treated with 150 and 600 mg/kg/day *C. longa*, or 30 and 120 mg/kg/day curcumin after PQ exposure period for 16 days. Total and differential white blood cells (WBC) and oxidative markers were measured both in bronchoalveolar lavage (BALF) and blood at the end of the study.

**Results:**

Total and differential WBC counts as well as malondialdehyde (MDA) level were significantly increased but total thiol content and the activities of catalase (CAT) and superoxide dismutase (SOD) were reduced in both the BALF and blood of the PQ group in comparison with the control group (p<0.05 to p<0.001). Both doses of *C. longa* and curcumin diminished MDA level, total and differential WBC counts in the blood and BALF but increased CAT and SOD activities in both of them compared to PQ group (p<0.05 to p<0.001). The effects of *C. longa* and curcumin high dose on most variables were markedly more than low dose (p<0.05 to p<0.001). Furthermore, the effects of curcumin on some variables were markedly more than* C. longa* (p<0.05 to p<0.001).

**Conclusion:**

Both *C. longa* and curcumin improved PQ-induced systemic and lung inflammation and oxidative stress, but the effect of curcumin was more prominent.

## Introduction

Agrochemicals such as paraquat (PQ) have widely been used in agriculture. PQ is a herbicide (Dinis-Oliveira et al., 2008 ▶) that might induce a serious hazard to human health (Chen et al., 2009 ▶; Kim et al., 2008 ▶) and PQ poisoning severity is dependent on the doses used (de Maglia et al., 2000 ▶). The possible mechanisms of PQ toxicity is inducing inflammation and oxidative stress by the generation superoxide anion (Hu et al., 2019 ▶) . PQ poisoning can leads to the disruption of nicotinamide adenine dinucleotide phosphate (NADPH) (Morán et al., 2010 ▶).

In the traditional medicine of southeast Asian countries such as India and China,* Curcuma longa* L. *(C. longa)* has been used for the treatment of common cold and asthma (Debjit Bhowmik et al., 2009 ▶). *C. longa* also, is used as a functional food and a spice in food preparation (Kocher et al, 2015 ▶). Curcumin (diferuloylmethane) (3–4%) is the main active constituent of the plant, which is responsible for its vibrant yellow color, consist of curcumin I, curcumin II and curcumin III (Kumar et al., 2011 ▶).

Different studies showed *C. longa* anti-inflammatory, anti-asthmatic (Chen et al., 2015 ▶; Shakeri et al., 2017 ▶), relaxant (Emami et al., 2017 ▶), antioxidant (Srinivas et al., 1992 ▶) and immunomodulatory effects by modulating the balance of regulatory T cells (Tregs) /T-helper (Th) in a mouse model of asthma (Shin et al., 2015 ▶).

Curcumin also showed anti-inflammatory (Jurenka, 2009 ▶), antioxidant (Trujillo et al., 2013 ▶), hepatoprotective (Koffler et al., 2000), relaxant (Emami et al., 2017 ▶), and anti-asthma properties (Shakeri et al, 2017 ▶; Boskabady et al., 2018 ▶) in various clinical and experimental studies. The anti-inflammatory, antioxidant, and immunomodulatory properties of *C. longa* and curcumin were reviewed recently, which documented their modulatory effects on various inflammatory mediators, cytokines and oxidant markers (Chainani-Wu, 2003 ▶; Memarzia et al., 2021 ▶). 

In the present study, the effects of *C. longa* and curcumin on PQ-induced lung and systemic inflammation and oxidative stress in rat were examined.

## Materials and Methods

**Animal and groups**

Thirty Wistar rats (male, weighted 200–250 gr) were purchased from the Faculty of Medicine animal house, Mashhad University of Medical Sciences (MUMS) and were maintained in plexiglass cages with free access to food and water, 22±2°C, humidity of 54±2%, and 12 hr light/dark cycle during the experimental period. The ethics committee of Mashhad University of Medical Sciences approved the animal experiments (IR.MUMS.MEDICAL.REC.1397.148). 

**Table 1:** Various studied groups, their exposing to saline or paraquate and treatment protocol

groups	Exposure	Treatment	Abbreviation
Control	Aerosol of saline	-	C
Paraquat	Aerosol of paraquat, 54 mg/m^3^	-	PQ
*C. longa*	“	150 mg/kg/day	Cl-L
	“	600 mg/kg/day	Cl-H
Curcumin	“	30 mg/kg/day	Cu-L
	“	120 mg/kg/day	Cu-H

Animals in control (C) group were exposed to normal saline and to paraquat (PQ, Sigma Aldrich Co, China,) aerosol (with dose of 54 mg/m3) in PQ group, (every other day, 8 times, 30 min in each time), (Burleigh-Flayer & Alarie, 1987) during 16 days. PQ or saline aerosols were delivered to head box of animal dimensions 15×18×30 cm (Amin et al., 2021). Four groups were treated with 150 and 600 mg/kg/day *C. longa* extract (Shakeri et al., 2017) or 30 and 120 mg/kg/day curcumin (Shakeri et al., 2018) for 16 days by gavage after the end of PQ exposure period (Heydari et al., 2019) (n=5 in each group). *C. longa* and curcumin were administered by gavage.

**Figure 1:**
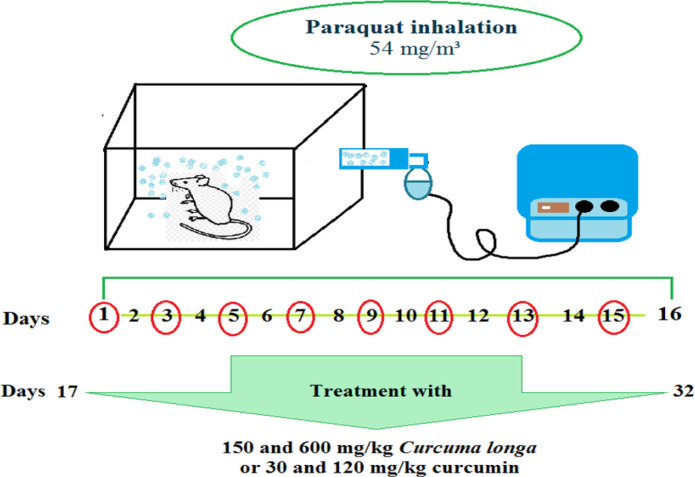
Protocol of animal’s exposure to PQ (54 mg/m^3^) aerosol and treatment with the extract of *Curcuma longa* and curcumin

Rats were randomly allocated to six groups (n=5 in each group) as described in Table 1. The method of animal exposure to PQ and saline aerosols as well as the method of treating animal to *C. longa* and curcumin were briefly described in the legend of Figure 1 (Amin et al., 2021 ▶).

**Plant and extract**

*C. longa* rhizomes (100 gr) were homogenized with ethanol 96% (Sigma-Aldrich Chemical Co., St. Louis, MO, USA) at a ratio of 1:10 (plant: ethanol) at 37°C for 3 days and occasionally shook. The extract was filtered and concentrated (under reduced pressure at 45°C in an Eyela. Heidolph, Schwabach, Germany) rotary evaporator as described previously ( Shakeri F, Boskabady MH., 2017 ▶). The extract yield was 15% (1.5 gr out of 10 gr rhizomes powder). Qualitative and quantitative determination of curcumin in the extract was also described in our previous study (Shakeri et al., 2017 ▶).

**Blood and bronchoalveolar lavage fluid preparation**

At the end of the treatment period, the animals were anesthetized by intraperitoneal (i.p.) injection of 1.6 gr/kg of urethane. The blood samples were taken from the heart and one ml of the blood samples were dispensed into the anti-coagulant containing for WBC counting. For measurement oxidative stress markers, the serum of the remaining blood samples was prepared and stored at −70°C.

Bronchoalveolar lavage fluid (BALF) was prepared as follows, after removal of the lungs from the chest BALF was obtained by washing the right lung with 1 ml normal saline for five times (total 5 ml) through tracheal cannula. The BALF was then centrifuged at 2500 rpm at 4°C for 10 min (Saadat et al., 2020 ▶). Cellular deposition was used for total and differential WBC count, and the collected supernatant was stored at −70°C for measurement of oxidative stress markers.

**Counting of total and differential white blood cell (WBC)**

Total WBC in the BALF and blood was counted using a hemocytometer (in a Burker chamber) in duplicate. From the cell pellet of the BALF and blood sample, a smear was prepared and stained with Wright-Giemsa for the differential WBC count. Differential MBC counts were then determined according to standard morphologic protocol under the light microscope (Saadat et al., 2019 ▶).

**Oxidant markers measurement**

The level of MDA and activities of CAT and SOD were measured according method described in our previous study (Saadat et al., 2019 ▶).

**Statistical analysis**

The results were compared among different groups using one-way analysis of variance (ANOVA) and Tukey’s multiple comparison test. Data were shown as mean±SEM. The value of p<0.05 was considered as the level of statistical significance.

## Results

**The results of WBC (total and differential)**

**The effects of paraquat**

**Figure 2:**
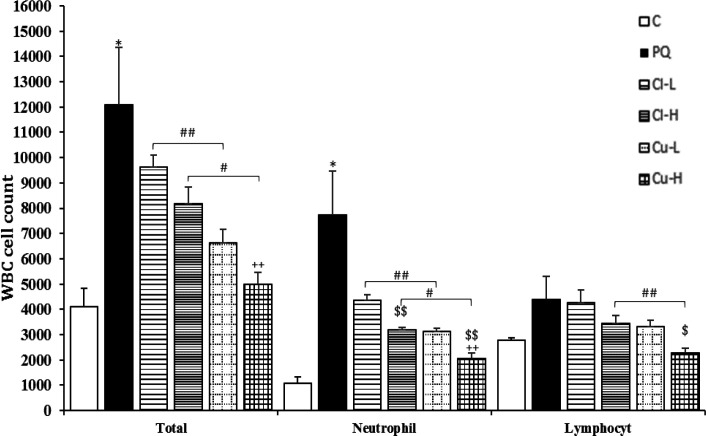
White blood cells (WBC, total and differential) counts in blood of the control group (C), group exposed to 54 mg/m^3^ paraquat aerosol (PQ), and groups exposed to PQ and treated with 150 and 600 mg/kg/day *C. longa* or 30 and 120 mg/kg/day curcumin (Cl-L, Cl-H, Cu-L and Cu-H respectively), (in each group, n = 5). *p<0.05 compared to the control group; +p<0.05 and ++p<0.01 compared to the PQ group; $p<0.05 and $$p<0.01 comparison between two doses of *C. longa*, and curcumin; # p<0.05 and ## p<0.01, comparison between *C. longa* and curcumin. The results are shown as mean±SEM. One-way ANOVA and Tukey’s test was applied for comparisons among different groups

**Figure 3:**
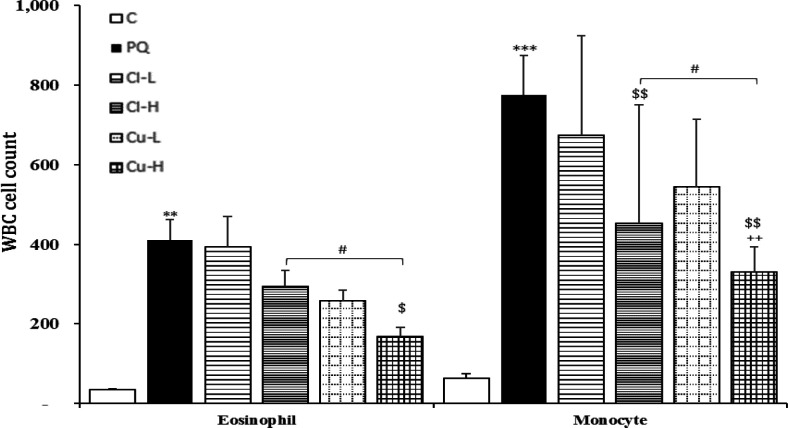
Eosinophil and monocyte counts in white blood cells (WBC) in blood of the control group (C), group exposed to 54 mg/m^3^ paraquat aerosol (PQ), and groups exposed to PQ and treated with 150 and 600 mg/kg/day *C. longa* or 30 and 120 mg/kg/day curcumin (Cl-L, Cl-H, Cu-L and Cu-H respectively), (in each group, n=5). **p<0.01 and ***p<0.001 compared to the control group; ++p<0.05, compared to the PQ group; $$p<0.01 comparison between two doses of *C. longa*, and curcumin; #p<0.05, comparison between *C. longa* and curcumin. The results are shown as mean±SEM. One-way ANOVA and Tukey’s test was applied for comparisons among different groups

**Figure 4:**
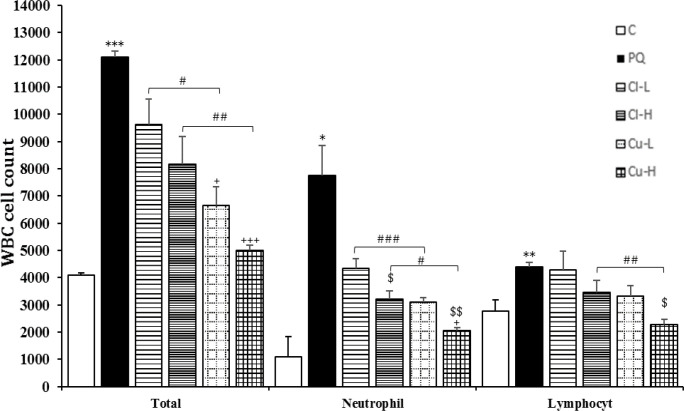
Total white blood cells (WBC), neutrophil and lymphocyte counts in the bronchoalveolar lavage fluid (BALF) of control group (C), group exposed to 54 mg/m^3^ paraquat aerosol (PQ), and groups exposed to PQ and treated with 150 and 600 mg/kg/day *C. longa* or 30 and 120 mg/kg/day curcumin (Cl-L, Cl-H, Cu-L and Cu-H respectively), (in each group, n=5). *p<0.05, **p<0.01, and ***p<0.001 compared to the control group; +p<0.05, +++p<0.001 compared to the PQ group; $p<0.05, $$ p<0.01 comparison between two doses of *C. longa*, and curcumin; #p<0.05, ## p<0.01, and ### p<0.001 comparison between *C. longa* and curcumin. The results are shown as mean±SEM. One-way ANOVA and Tukey’s test was applied for comparisons among different groups

**Figure 5:**
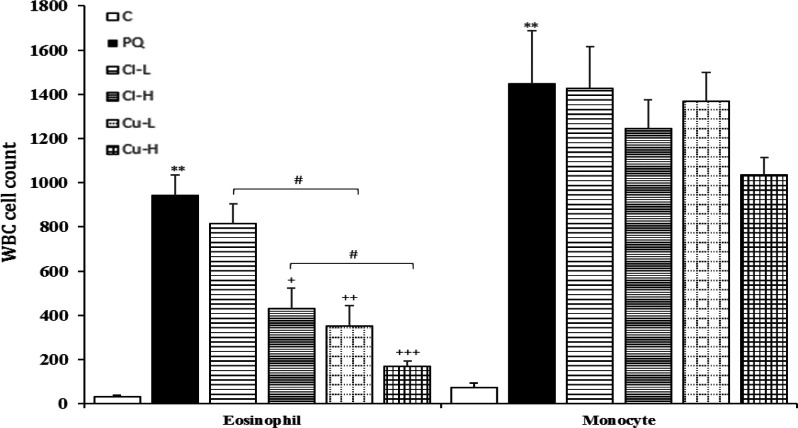
Eosinophil and monocyte counts in the BALF of control group (C), group exposed to 54 mg/m^3^ paraquat aerosol (PQ), and groups exposed to PQ and treated with 150 and 600 mg/kg/day *C. longa* or 30 and 120 mg/kg/day curcumin (Cl-L, Cl-H, Cu-L and Cu-H respectively), (in each group, n=5). **p<0.01 and ***p<0.001 compared to the control group; +p<0.05, ++p<0.01, and +++p<0.001 compared to the PQ group; #p<0.05, comparison between *C. longa* and curcumin. The results are shown as mean±SEM. One-way ANOVA and Tukey’s test was applied for comparisons among different groups

Exposure of rats to inhaled PQ (54 mg/m^3^) resulted in a significant increase in total and all differential WBC both in the BALF and the blood except lymphocyte count in the blood (p<0.05-p<0.001, Figures 2-5).

**The effects of** ***C. longa*** **extract treatment**

Treatment with high dose of the *C. longa *extract significantly decreased neutrophil count in the blood and the BALF and eosinophil count in the BALF (p<0.05 for all cases), (Figures 2-5). The effect of high dose of the extract on neutrophil count both in the blood and in the BALF was significantly higher than its low dose (p<0.05 for the BALF and p<0.01 for the blood), (p<0.05 for the BALF and p<0.01 for the blood).

In the blood, the effect of the extract high dose on monocyte count was significantly higher than its low dose. However, in the BALF there were not significant differences between two doses of the *C. longa *extract on that of WBC (total and differential counts (Figure 2). 

**The effects of curcumin treatment**

Total WBC and neutrophil counts both in the blood and the BALF as well as eosinophil count in the BALF were significantly reduced in groups treated with 

both doses of curcumin (p<0.05- p<0.001). Lymphocyte counts both in the BALF and 

the blood, and eosinophil count in the blood were also significantly reduced in the group treated with high dose of the curcumin (p<0.05 for all cases). Monocyte counts were also significantly reduced in the groups treated with high dose of the curcumin and *C. longa* in the blood (p<0.01, Figures 2-5). The effects of high dose of curcumin on differential counts of all WBC subtypes (both in the blood and in the BALF) were significantly higher than those of low dose, except monocyte count in the BALF (p<0.05-p<0.01, Figures 2-5).

**The results of oxidative stress markers**

**The effects of paraquat**

**Figure 6:**
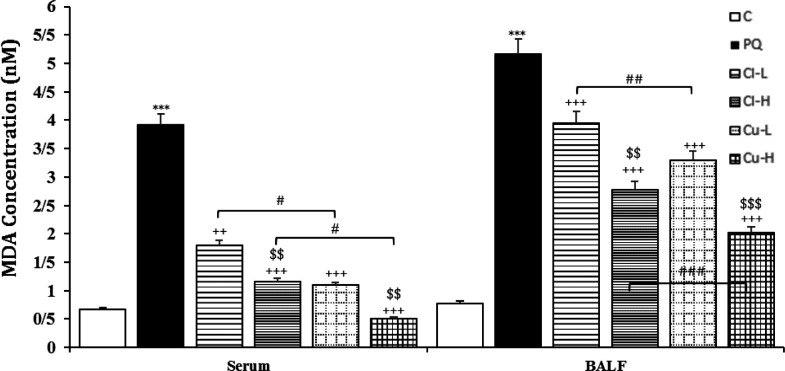
Malondialdehyde (MDA) level in the serum and the BALF of the control group (C), group exposed to 54 mg/m^3^ paraquat aerosol (PQ), and groups exposed to PQ and treated with 150 and 600 mg/kg/day *C. longa* or 30 and 120 mg/kg/day curcumin (Cl-L, Cl-H, Cu-L and Cu-H respectively), (in each group, n=5). ***p<0.001 compared to the control group; ++p<0.01 and +++p<0.001 compared to the PQ group; $$ p<0.01 and $$$, comparison between two doses of *C. longa*, and curcumin; # p<0.05, ## p<0.01, and ### p<0.001, comparison between *C. longa* and curcumin. The results are shown as mean±SEM. One-way ANOVA and Tukey’s test was applied for comparisons among different groups

**Figure 7:**
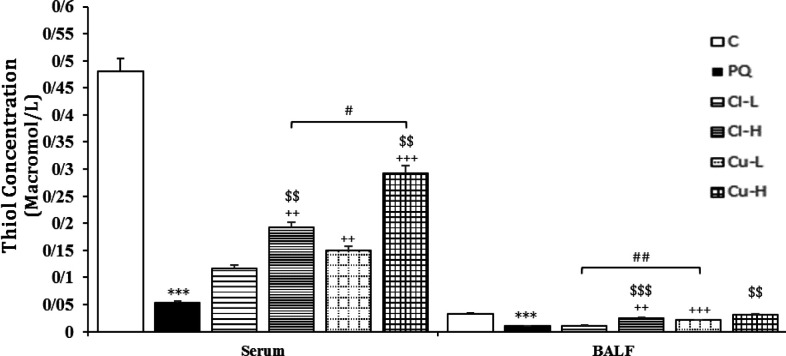
Thiol level in the serum and the BALF of the control group (C), group exposed to 54 mg/m ^ 3^ paraquat aerosol (PQ), and groups exposed to PQ and treated with 150 and 600 mg/kg/day *C. longa* or 30 and 120 mg/kg/day curcumin (Cl-L, Cl-H, Cu-L and Cu-H respectively), (in each group, n=5). ***p<0.001 compared to the control group; ++p<0.01 and +++p<0.001 compared to the PQ group; $$ p<0.01 and $$$ p<0.001 comparison between two doses of *C. longa*, and curcumin; # p<0.05 and ## p<0.01, comparison between *C. longa* and curcumin. The results are shown as mean±SEM. One-way ANOVA and Tukey’s test was applied for comparisons among different groups

**Figure 8:**
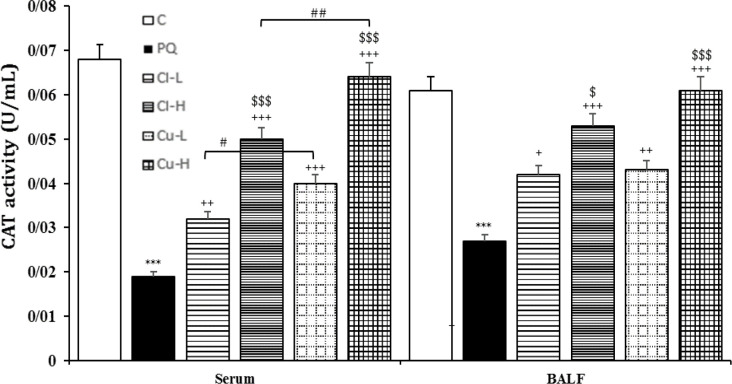
Catalase (CAT) level in the serum and the BALF of the control group (C), group exposed to 54 mg/m^3^ paraquat aerosol (PQ), and groups exposed to PQ and treated with 150 and 600 mg/kg/day *C. longa* or 30 and 120 mg/kg/day curcumin (Cl-L, Cl-H, Cu-L and Cu-H respectively), (in each group, n=5). ***p<0.001 compared to the control group; +p<0.05, ++p<0.001, and +++p<0.001, compared to the PQ group; $ p<0.05 and $$$ p<0.001, comparison between two doses of *C. longa*, and curcumin; #p<0.05 and ##p<0.01, comparison between *C. longa* and curcumin. The results are shown as mean±SEM. One-way ANOVA and Tukey’s test was applied for comparisons among different groups

**Figure 9:**
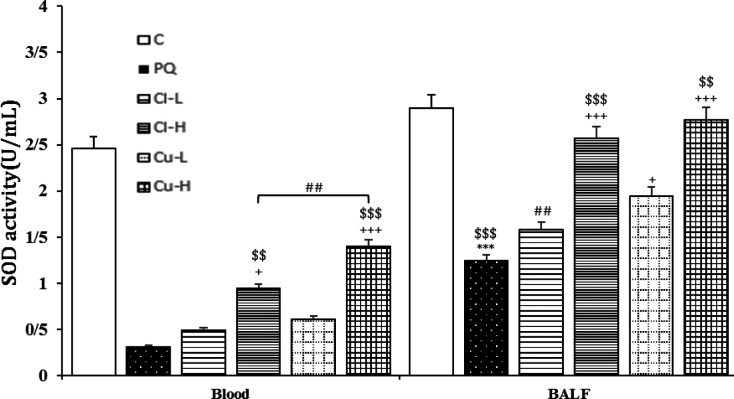
Superoxide dismutase (SOD) concentration in the serum and the bronchoalveolar lavage fluid (BALF) of the control group (C), group exposed to 54 mg/m^3^ paraquat aerosol (PQ), groups exposed to PQ and treated with 150 and 600 mg/kg/day *C. longa* or 30 and 120 mg/kg/day curcumin (Cl-L, Cl-H, Cu-L and Cu-H respectively), (in each group, n=5). ***p<0.001 compared to the control group; +p<0.05 and +++p<0.001, compared to the PQ group; $$p<0.01, $$$p<0.001 comparison between two doses of *C. longa*, and curcumin; ## p<0.01, comparison between *C. longa* and curcumin. The results are shown as mean±SEM. One-way ANOVA and Tukey’s test was applied for comparisons among different groups

The level of MDA was significantly increased, but those of CAT, SOD and thiol were decreased due to exposure of rats to PQ inhalation (p<0.001 for cases, Figures 6-9).

**The effects of** ***C. longa*** **extract treatment**

Both doses of the extract decreased the level of MDA and increased the thiol level. In contrast, CAT increased in treating animals with high dose of extract both in the serum and BALF, as well as SOD activities were increased in the high dose of serum and the BALF (p<0.05-p<0.001, Figures 6-9). The effects of high dose of the extract on all oxidative stress markers both in the blood and in the BALF were significantly 

higher than those of low dose (p<0.05- p<0.001, Fgures 6-9).

**The effects of curcumin treatment**

Treatment with both doses of the curcumin decreased the levels of MDA, while increased CAT both in the serum and the BALF (p<0.05 for CAT in the BALF and p<0.001 for other cases). The effects of high dose of curcumin on all oxidative stress markers, both in the serum and in the BALF were markedly higher than those of low dose (p<0.01-p<0.001, Figures 6-9).

**Comparison between the effects of** ***C. longa*** **and curcumin**

The effects of high dose of curcumin on total and differential WBC in the BALF and the blood except monocyte in the BALF were markedly higher than those of the high 

dose of the extract (p<0.05-p<0.001). The effects of low dose of curcumin on total WBC and neutrophil both in the blood and the BALF and on eosinophil in the BALF 

were also higher than those of low dose of the extract (p<0.05-p<0.001, Figures 2-5). Treatment with high dose of curcumin improved MDA levels, both in the serum and the BALF, and the serum CAT activity was significantly higher than those of the *C. longa* extract (p<0.5- p<0.001). The effects of high dose of curcumin on the serum thiol content and SOD activity, and its low dose on the BALF thiol level were also significantly higher than corresponding doses of the extract (p<0.05-p<0.001, Figures 5-9).

## Discussion

In this study, the effects of the *C. longa* ethanolic extract and curcumin as the main constituent of the plant, on the total and differential WBC as well as oxidant markers (MDA, thiol, SOD, and CAT) on a rat model of PQ-induced lung and systemic toxic changes were investigated.

Sixteen days exposure of rats to PQ aerosol (54 mg/m^3^) inhalation significantly increased the total and differential WBC counts as well as MDA level, but CAT, SOD and thiol levels were significantly decreased both in the blood and the BALF which were in line with previous findings (Amin et al., 2021 ▶). However, treatment with the extract of the plant and curcumin, improved WBC (total and differential) counts as well as oxidant markers (MDA, thiol, SOD, and CAT) both in the blood and the BALF.

Experimental models of PQ toxicity have been proposed as an oxidant-initiated lung and systemic degenerative and inflammatory lesions (Amin et al., 2021 ▶; Bus & Gibson, 1984 ▶). PQ generates the superoxide anion that resulted in production of more reactive oxygen and toxic oxidative stress biomarkers, which confirmed the present study findings as lung and systemic elevation of MDA and decrease in thiol, SOD, and CAT. Therefore, antioxidant therapy has been considered as a beneficial strategy in the healing and prevention of the toxic effects of PQ (Suntres, 2002 ▶). The antioxidant properties of *C. longa* and curcumin against asthma model induced systemic and lung oxidative stress were indicated (Shakeri et al., 2017 ▶). In addition, several antioxidant effects including reduction of MDA, CK-MB, LDH activities, and NO metabolites and elevation of SOD, CAT, GSH, GPX and GR have been suggested for *C. longa* in various toxic and inflammatory experimental models and clinical trials (Memarzia et al., 2021 ▶).

The findings of inflammation and oxidative stress may show a cause and effect relationship. Elevation of ROS might cause lung and systemic inflammation, which increase and activate eosinophils, neutrophils, monocytes, and macrophages for generation of more ROS (Biswas, 2016 ▶; Shakeri et al., 2017 ▶). In this study, PQ-induced oxidative stress was in line with total and differential WBC changes.

The results of this study showed that the higher doses of *C. longa* and curcumin were more effective against PQ-induced elevation of WBC (total and differential) and MDA level, and also the reduction of thiol content and antioxidant enzymes in blood and BALF. In most of toxic and inflammatory experimental models the effective dose of *C. longa* or its extract was more than 200 mg/kg and the ethanolic extract was more effective than aqueous extract or *C. longa *powder (Memarzia et al., 2021 ▶), which confirmed the result of this study. Moreover, the dose dependent protective effects of both *C. longa* and curcumin on WBC and oxidative stress marker were indicated previously in ovalbumin-induced asthma rat model (Shakeri et al., 2017 ▶). The present study findings showed that the higher dose of curcumin (120 mg/kg) had more therapeutic potency than higher dose of *C. long *(600 mg/kg). Therefore, the antioxidant and anti-inflammatory effects of *C. long* might be attributed to curcumin, which is the main constituents of *C. longa*. In previous studies the comparative effects of *Curcuma longa* and curcumin with those of dexamethasone were shown (Shakeri et al., 2017 ▶, Boskabady MH et al., 2021 ▶) therefore, in the present study a positive group was not performed.

Regarding to the safety of *C. longa* even at high dose and many document addressing its anti-inflammatory and antioxidant effects in clinical trials (Memarzia et al., 2021 ▶), thus the plant might be a good candidate to prevent PQ poisoning in clinics.

*C. longa* and curcumin as the main constituent of the plant improved inhaled PQ- induced lung and systemic oxidative stress and inflammation but the effect of curcumin was more prominent. These findings indicate a potential therapeutic effect of *C. longa* and specially curcumin in inhaled PQ-induced lung and systemic oxidative stress and inflammation.

## Conflicts of interest

The authors have declared that there is no conflict of interest.
